# A qualitative study of design stakeholders’ views of developing and implementing a registry-based learning health system

**DOI:** 10.1186/s13012-020-0976-1

**Published:** 2020-03-06

**Authors:** Mary Dixon-Woods, Anne Campbell, Trillium Chang, Graham Martin, Alexandros Georgiadis, Veronica Heney, Sarah Chew, Aricca Van Citters, Kathryn A. Sabadosa, Eugene C. Nelson

**Affiliations:** 1 THIS Institute, Department of Public Health and Primary Care, University of Cambridge, Cambridge Biomedical Campus, Clifford Allbutt Building, Cambridge, CB2 0AH UK; 2 The NIHR Health Protection Research Unit in Healthcare-Associated Infections and Antimicrobial Resistance, Imperial College, Hammersmith Campus, London, W12 0NN UK; 3 Stanford Law School, 559 Nathan Abbott Way, Stanford, CA 94305 USA; 4 ICON plc, Cambridge Biomedical Campus, Clifford Allbutt Building, Cambridge, London, CB2 0AH UK; 5 Wellcome Centre for Cultures and Environments of Health, University of Exeter, Queens Drive, Exeter, EX4 4PZ UK; 6 Social Science Applied to Healthcare Improvement Research (SAPPHIRE) Group, Department of Health Sciences, University of Leicester, George Davies Centre, University Road, Leicester, Leicester, LE1 7RH UK; 7 The Dartmouth Institute for Health Policy & Clinical Practice, Dartmouth College, Level 5, WTRB, 1 Medical Center Drive, Lebanon, NH 03756 USA; 8 Cystic Fibrosis Foundation, 4550 Montgomery Ave., Suite 1100 N, Bethesda, MD 20814 USA

**Keywords:** Quality improvement, Replication, Program theory, Qualitative, Co-production, Registries, Learning health systems, Cystic fibrosis

## Abstract

**Background:**

New opportunities to record, collate, and analyze routine patient data have prompted optimism about the potential of learning health systems. However, real-life examples of such systems remain rare and few have been exposed to study. We aimed to examine the views of design stakeholders on designing and implementing a US-based registry-enabled care and learning system for cystic fibrosis (RCLS-CF).

**Methods:**

We conducted a two-phase qualitative study with stakeholders involved in designing, implementing, and using the RCLS-CF. First, we conducted semi-structured interviews with 19 program personnels involved in design and delivery of the program. We then undertook 11 follow-up interviews. Analysis of interviews was based on the constant comparative method, supported by NVivo software.

**Results:**

The organizing principle for the RCLS-CF was a shift to more partnership-based relationships between patients and clinicians, founded in values of co-production, and facilitated by technology-enabled data sharing. Participants proposed that, for the system to be successful, the data it collects must be both clinically useful and meaningful to patients and clinicians. They suggested that the prerequisites included a technological infrastructure capable of supporting data entry and joint decision-making in an accessible way, and a set of social conditions, including willingness from patients and clinicians alike to work together in new ways that build on the expertise of both parties. Follow-up interviews highlighted some of the obstacles, including technical challenges and practical constraints on refiguring relationships between clinicians and patients.

**Conclusions:**

The values and vision underlying the RCLS-CF were shared and clearly and consistently articulated by design stakeholders. The challenges to realization were often not at the level of principle, but were both practical and social in character. Lessons from this study may be useful to other systems looking to harness the power of “big data” registries, including patient-reported data, for care, research, and quality improvement.

Contributions to the literature

- The concept of a learning system has been enthusiastically embraced in healthcare, but relatively few real-life examples exist and even fewer have been evaluated.
- It is important to articulate the assumptions and values that underlie learning systems and to characterize the challenges they may face.
- Interviews with design stakeholders involved in developing and implementing a US learning system showed that they were commited to realizing authentic partnerships between patients and clinicians, facilitated by a shared clinical dashboard fed with data by both parties.
- They also identified technical and social challenges to achieving this ideal, offering practical learning for others seeking to implement learning systems.

## Background

Supporting patients with chronic conditions in ways that fully respect their needs, values, capabilities, and priorities remains a challenge for health systems [1, 2]. At the same time, there is a growing requirement for better data to support improvement in care quality and to facilitate research that is relevant to the needs and priorities of patients and clinicians [3]. One promising approach to addressing these imperatives lies in the concept, originally proposed by the Institute of Medicine, of the learning health system [4, 5]. Leveraging the capabilities of electronic health records and other digital resources, learning health systems seek to support collaborative healthcare choices of patients and clinicians, generate new knowledge as an “ongoing, natural by-product of the care experience” [6], and facilitate improvements in quality, safety, and value [7].

Though articulated in various forms, the underlying concept is straightforward: harness the power of data and analytics to learn from every patient, and feed the knowledge of “what works best” back to clinicians, public health professionals, patients, and other stakeholders to create cycles of continuous improvement [8].

Despite enthusiasm for the principles of a learning health system, few real-life examples exist [9, 10] such that “a learning health system remains an aspiration rather than an achievement” [3]. The risk is that, unless early models are studied [11], learning health systems may not mature to the point where their ambitions can be delivered. In this article, we report a study of the views of design stakeholders of a program known as “Enabling Uptake of a Registry-Supported Care and Learning System in the US” (RCLS) for people with cystic fibrosis (CF). Funded by the Robert Wood Johnson Foundation and the Cystic Fibrosis Foundation, the RCLS-CF was led by a team from The Dartmouth Institute for Health Policy & Clinical Practice (TDI) in New Hampshire, USA, with support from the US Cystic Fibrosis Foundation, and advice from Cincinnati Children’s Hospital Medical Center, Cincinnati, OH, USA, and the Karolinska Institutet in Stockholm, Sweden.

The RCLS-CF is an important example for several reasons. First, it is based on a defined patient population rather than a single organization. Second, it combines data from a patient registry of clinical measures with newly recorded patient-reported data to feed a distinctive electronic dashboard that can be viewed jointly by patients and clinicians and used as a basis for collaborative shared review, planning, and decision-making [12]. Third, the data can be repurposed for research and service improvement. Fourth, the RCLS-CF explicitly seeks to learn from the achievements and experiences of other programs that share some of the characteristics of the learning system approach. In particular, it builds on the work of Swedish and American programs that seek to facilitate real-time input of data by patients [13] [14, 15], joint review of data by clinicians and patients during clinic appointments [16], and co-design of systems by patients, families, clinicians, and researchers [17–19].

We conducted a qualitative study of the RCLS-CF learning system to examine perspectives of the health professional design stakeholders, seeking in particular to articulate their major assumptions about the essential features of the program and what would be required to make it work [20–24].

## Methods

We conducted the study in two phases. In early 2016, the first phase of the study involved semi-structured interviews with health professional stakeholders involved in designing, advising, implementing, and evaluating the RCLS-CF. The program director invited 20 health professionals (i.e., front line interdisciplinary team members and other professionals closely engaged in the design of the project) to take part in a confidential interview. These personnel included program designers [5], program advisors [7], pilot site leaders [5], and program sponsors/funders [3]. Purposive sampling was used to ensure a balance of individuals such as site leaders to avoid over sampling, but otherwise, all the major professional stakeholders were invited to take part. Interviews were conducted by telephone or Skype with 19 participants shortly after the program began. One individual (a program advisor) was not interviewed owing to scheduling difficulties.

The second phase of the study sought to deepen understanding of the findings generated in the first phase and to identify any changes that might have occurred over time in light of experience. In spring 2018, the project director invited 17 of the original interviewees to participate in a further interview (excluding only three who were no longer involved in the program). Three declined to be interviewed due to lack of engagement in the program since its inception; three did not respond to the interview request. In total, 11 stakeholders were re-interviewed by phone; they included a program designer [1], program advisors [4], pilot site leaders [4], and program sponsors/funders [2].

The interview guides in both phases were informed by the TiDIER [25] framework for describing interventions. They included questions about what participants perceived to be the essential components of the program, how they were expected to work, and the contextual factors perceived to have impact on the successful replication of the program. The first round of as interviews was carried out by AC; the follow-up interviews were carried out by TC. AC is a doctorally trained health service researcher; TC is prepared in public health, who conducted the study in partial fulfillment of the requirements of a master’s dissertation. Neither had any relationship with the program team. All interviews were digitally recorded. Interview recordings were transcribed verbatim. Transcripts were anonymized to remove any identifying details. Full transcripts were not shared with the RCLS-CF team.

Analysis of interviews was based on the constant comparative method [26], using initial sensitizing constructs from the TIDieR framework to examine the transcripts. New codes were iteratively developed as analysis continued, and higher order thematic categories were developed. Trustworthiness was enhanced by the involvement of different team members in analysis and refinement of the themes, and through the use of NVivo 11 software to manage the coding and analysis process and facilitate a systematic and transparent approach to coding. Credibility was enriched by checks to ensure that the themes were adequately comprehensive, with no relevant data excluded or irrelevant data included [27]. Credibility was also secured through sharing of the themes with members of the authorship team who were also directly involved with the RCLS-CF (AVC, KAS, ECN), who provided further insights derived from their own experience of the program.

Both phases of the study were approved by the Dartmouth College Committee for the Protection of Human Subjects (Study: 00028656, approved 23 September 2015). All participants gave written informed consent.

## Results

Interviews with participants allowed characterization of the key elements of the RCLS-CF learning system (Table 1) as understood by the health professional design stakeholders. In the account that follows, we examine, first, the vision and foundational concepts of the learning system that were identified by participants; second, participants’ views of the technical prerequisites; third, their views of the social conditions necessary for program implementation; and finally, challenges in implementation. Except where relevant, we do not draw contrasts between the first part of data collection and the second, instead presenting findings that endured between the two phases. We identify interviews as arising from the first phase of data collection as 1-, and from the second phase as 2-, in the tags that follow data excerpts.

**Table 1:** Summary of key components of the registry-enabled learning system (RCLS-CF)

Topic	Core idea
Program origins	• The program is informed by and to some extent modeled on existing initiatives • The program is informed by two important ideas: the learning health system and co-production
Design principles	• A shift to more partnership-based relationships between patients and clinicians can be facilitated by new forms of technology-enabled data-sharing • Data generated by patients and clinicians can be repurposed for other uses, including research and service improvement • The key stakeholders must be involved in co-design of RCLS-CF • The data collected must be meaningful to patients and clinicians and reflect their priorities
Design constraints	• The technology will need to be smooth, effective, and time-efficient to use • Ensuring security and privacy of data will be essential
Implementation tactics	• *Program Scope*: The RCLS-CF program will focus primarily on developing the dashboard that reflects patient’s goals, treatments, and outcomes • Securing universal support from clinicians and patients may be challenging • The project is complex and may generate some frictions
Risk mitigation	• The program may create additional burdens and risks for patients • The program may create or be influenced by forms of inequity

The information in this table is based on stakeholder interviews with RCLS-CF program designers, advisors, leaders from pilot sites, and program sponsors/funders

### Design stakeholders’ views of tthe foundations of RCLS-CF: co-production and its transformational potential

**Fig. 1:**
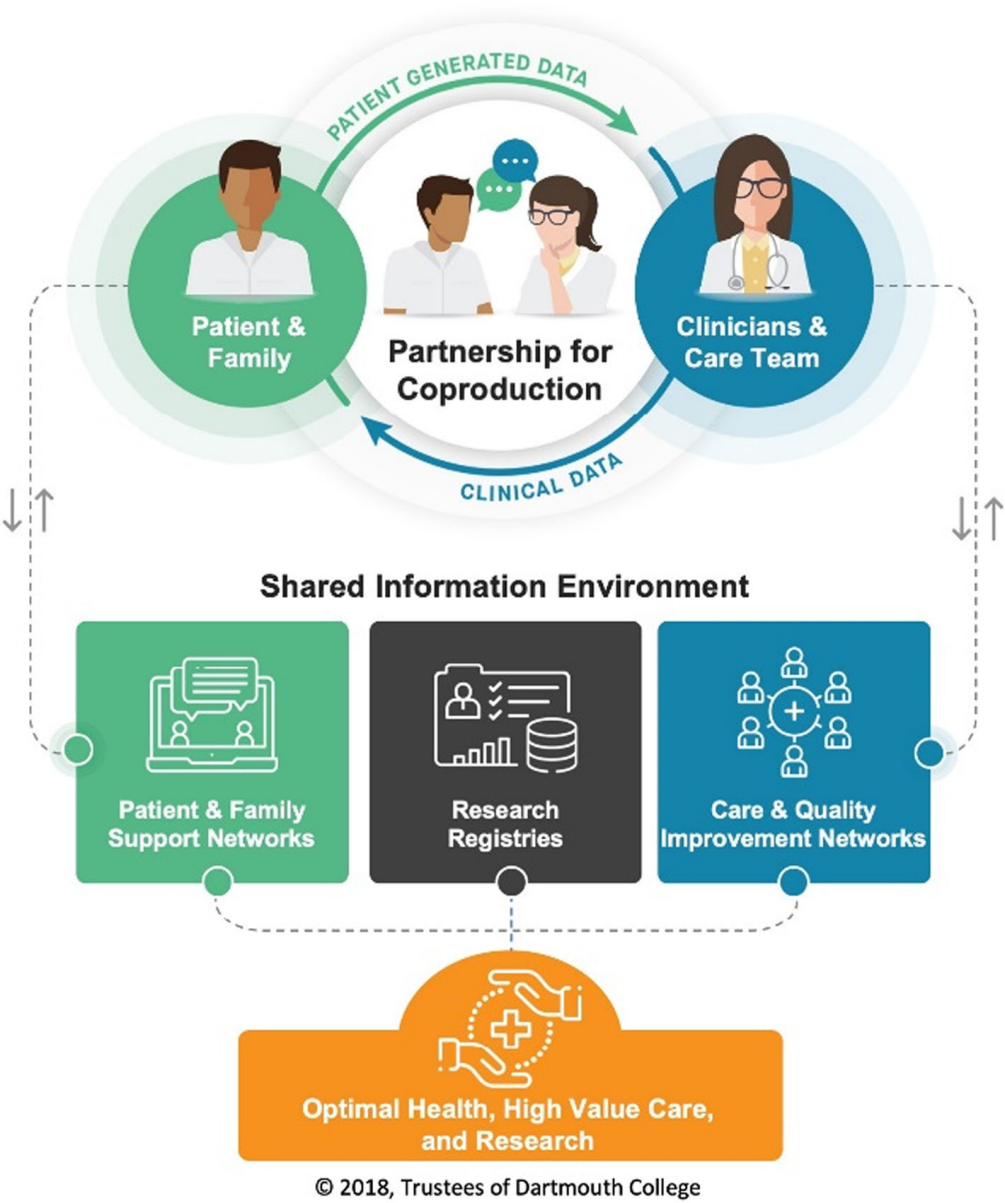
Conceptual model of the RCLS-CF program: linking people, information, and knowledge generation for better health

The program team developed a high-level conceptual model that sought to link people, information, and knowledge generation to achieve better health (Fig. 1), and this remained intact over time. In both parts of the study, participants expressed strong consensus on the goals of the program: to use new, digitally enabled forms of sharing data (both patient-generated and clinical) to change the dynamics of healthcare, with the ultimate aim of facilitating better partnerships between patients, families, clinicians, researchers, and healthcare improvers.

At the center of this technical infrastructure was a dashboard intended to put patient-reported and clinical data on an even keel, allowing patient and clinician to attend to both, to understand their interactions, and to adjust therapeutic regimes to optimize management of the condition in light of both patient and clinician priorities. In practical terms, the program viewed every clinical interaction and every patient input to the system as an opportunity to generate meaningful and usable knowledge that could improve individuals’ health outcomes and support decision-making, and could also potentially contribute to a knowledge base that could be used for scientific discovery and service improvement.

An especially prominent feature of participants’ accounts of the program in both phases was an emphasis on ideas of co-production [28]. As they saw it, co-production provided a value-based framework through which it would be possible to redefine the nature of care delivery and the relationship between patients and clinicians, enabling it to be more dialogical, more equitable, and more facilitative of shared decision-making.

Well the heart of it is the partnership for co-production. That’s kind of where the magic happens. (1-06 Sponsor/funder)

Participants described a vision where better conversations, grounded in patients’ priorities, would allow better care to be co-created. Achieving this would mean providing patients with the means to identify the metrics most important to them, to report on what was happening between clinic visits, to shape the agenda of visits, and to work with clinicians as equal partners.

The way that I see the basics of this, is that you know we are putting patient-reported data, […] and some of the clinically collected data, on the same playing field. (1-16 Advisor)

In the follow-up (phase 2) interviews, participants suggested thinking of the clinical encounter as involving two experts: the clinician, an expert in biomedical knowledge, and the patient, an expert in their own experience of living with a life-limiting illness. It was proposed that this reconfigured relationship would result in new forms of partnership.

The social relationship starts to often change when successful, that by creating this new information environment the ability to have a more equal relationship, where we have... discovered this phrase, two experts in the room, the patient is an expert on their own results and what seems to be working for them and how this...the disease and its treatment can fit into their life. The physician, clinician is the expert in what biomedical science might have to say about treating the diseases or disease or the conditions that the person has. So now we have two experts in the room, that’s new. (2-05 Designer)

Looking at the data together during clinic visits, and organizing discussions around it, was specifically intended to provide the basis for better conversations, shared decision-making, and, ultimately, better health outcomes for the individual patient.

I would say the aim is to really change the model of care to […] a model that was previously more traditional […] top-down, where the provider was more the decision maker, and […] the patient was more passive to a model that is more of a partnership between the patient and the, the patient, family, and the team. (1-12 Pilot Site Leader)

I think we’ll get much better outcomes if we engage patients in their own care. And part of that is eliciting what are the goals of care for the patient and then constructing a, sort of, shared decision making, you know, a goal informed by shared decision making. (2-03 Advisor)

The underlying assumption was that the registry-enabled approach would promote shared understanding of the data and therefore joint decision-making. The information generated by patients and clinicians could also be used for wider purposes in keeping with the ethos of the learning health system: the growing trove of data in the registry could also be used for research and for service improvement.

The learning health system is a wonderful idea, it is a really cool thing where you know every time people meet, any data that is gathered is then sort of hoovered up and turned into knowledge that is then applied to the point of care so that people can make better decisions. So you know the connection between the two is you know if you have these highly productive, co-produced clinical interactions, then the knowledge, the data that starts generating as a result of those interactions is somehow contributed to the greater pool of knowledge. (1-10 Advisor)

Consistent with these principles, a process of “co-design,” in which representatives of key groups had been involved in formulating and planning the program from an early stage, was seen as important to ensuring that the program was grounded in co-production and could address both technical and social challenges. Participants emphasized that co-design of the dashboard was both an ethical imperative and necessary to ensuring that the system would secure engagement.

We have a very active co-design process, we have representatives. I think of it as the lead team […] we have involved interdisciplinary clinical teams, including patients and family members in each of those five settings, they are the lead teams that are looking at this model that we are looking at, and coming to understand it, and helping to decide what those dashboards look like, what the information technology solution approach will be and the CF Foundation is at the table as well. (1-15 Advisor)

Participants consistently identified the key stakeholders in the project as the patients and their families, the care team, and the Cystic Fibrosis Foundation, as well as senior executives in individual organizations. Others included electronic health record vendors, specialist pharmacies, researchers, and regulators and payers.

So we have a coalition of patients, providers, researchers and industry participation. And that coalition of stakeholders makes it possible for all participating organizations to negotiate the values of the different stakeholders in participation. And it also makes it possible for the stakeholders to negotiate core data sets that have big importance for all stakeholders. So that organizational dimension is very important. (2-06 Advisor)

### Design stakeholders’ views of the technical prerequisites for the learning system

Participants emphasized that facilitating more co-produced care plans, informed by both clinical and patient-reported data, would need to be supported by a point-of-care dashboard that could be viewed in clinical consultations by patient and clinician together. By collecting and storing patient and clinical data from the CF Foundation Patient Registry together in the dashboard, the assumption was that patients would be able to reflect on the course of their disease, symptoms, and medical interventions and develop their own goals and share them with clinicians. This would require a means for patients to select measures that were important to them and to capture their own data on events (e.g., ability to participate in daily activities or exercise), symptoms occurring between clinic visits, and variations in their clinical status (e.g., weight, lung function) over time. Participants described how patient-reported and clinical data would feed the electronic dashboard and simultaneously supply the CF Foundation Patient Registry with patient-reported outcomes.

The three-year goal is much more specific and that is to take components of this larger model, and to make it possible to have a dashboard at the center of the model, that can be used at the point-of-care, when patients and clinicians get together, to help see how the person is doing. To help make decisions about how well the treatment plans are working, to help inform the conversation about the best next steps in the treatment plan, based on patient preference and evidence base. (1-15 Designer)

Participants identified that for these goals to be achieved, the technical infrastructure would need to meet several key requirements. In particular, it would need to be technically capable of supporting clinical care, research, and improvement; create an easy, non-frustrating way for patients to record data and make direct inputs to their own health records; help clinicians to record and view key clinical measures without irritating them or slowing them down; and enable both sets of data (patient-generated and clinician-generated) to be displayed visually for easy use by patients, families, and clinicians as part of routine visits.

The technology is the first part. Like you have to have solid technology that is really easy to use and you know, not buggy, and fast, and all those important things. (1-16 Advisor)

Probably the critical components [are] ... aggregating the medical data in a way that is easily digestible, and the patient-reported outcome measures, getting them, getting them in real time, organized in a way that’s easily digestible and available to all parties. (1-06 Sponsor/funder)

Participants described the need for an interface that was smooth and fast, where end-users could enter data in real time via multiple channels and enjoy flexibility in the types of data that could be entered. It also needed to avoid any double entry: participants emphasized the principle of “data in once.”

It needs to be today’s data is actually entered in the dashboard. So we can say how are you doing today and what are the things contributing and, you know, do we need to make changes. (1-19 Pilot Site Leader)

What I worry about is, it may create the need to do more data entry that is manual between all of these systems to get what we really want up and going. […] I think there are huge burdens between our current clinic [electronic medical record] system, our hospital system none of these are at the moment interoperable and that, I worry about where the burden of getting that data is going to fall if it doesn’t seamlessly flow with this project. (1-12 Pilot Site Leader)

In practice, multiple technical problems emerged in achieving the seamless, on-demand flow of data that the designers sought. By the phase 2 interviews, participants were stressing many of the challenges:

The platform we were using wasn’t real time, so you didn’t have today’s data there and you only had up till the previous visit’s data, and any discussion really centers around how are things going at today’s appointment, and where is your lung function today? So you absolutely have to have real time data and that’s crucial." (2-04 Site lead)

Although it’s workable in theory we didn't succeed in our four pilot sites in getting single sign- on and we did not succeed in getting two-way exchange of real time data. We could get...for example we couldn’t get real- time clinical results that are fundamental, FEV1 and body mass index, that was always lagged. So today’s values for body mass index and today’s value for lung function, FEV1, weren’t there. The historical dates were, data points were but not today’s," (2-02 Program designer)

During the initial interviews, participants reported that the program would need to work within, or alongside, current electronic healthcare record systems and have appropriate security protections and permissions for access. Participants consistently identified the complexity of the healthcare system in the USA as a challenge to the technical feasibility of the program, with interoperability a particular threat that was especially prominent in the second phase of interviews.

And the interoperability of data, from, if we want to produce a single dashboard for example, if we decide to do that then we’ll have to deal with interoperability challenges […] data in one system is not always defined the same as it is in another system […] so we have to make sure there’s clear mapping between, or from different systems on to the dashboard. (1-08 Designer)

And then the other part of the programme was to try and implement this through an electronic feature or system. But [pause] the system, electronic system, didn't work because we lacked the infrastructure ability to make it inter-operable with the charts" (2 – 03 Site Lead)

### Design stakeholders’ views of the social conditions necessary for program implementation

Though optimizing and resolving technical issues were seen as important to ensuring that the program could work, participants were also cognizant of wider contexts. They recognized that the program might not gain universal support and identified many social, cultural, and practical barriers that might prevent clinicians and patients from engaging with the program. Participants noted that not all people with CF would find the dashboards appealing or easy to use. Some patients might revert to passivity, preferring to simply listen and follow advice from clinicians rather than engaging as partners.

I think for some patients it will also be difficult in that patients like to view their doctor as knowing everything and the idea that doctors need to share for patients beyond the just regular questions that are asked may be difficult for some patients to come to terms with […] a patient may never expect a doctor to know like what is going on in the home. (1-09 Pilot Site Leader)

Feeding forward patient-reported information would, it was acknowledged, add to the burden of data collection for patients who already spent much time on self-care. Engagement might further be influenced by factors such as literacy, education, self-efficacy, and access to the relevant technology. It was suggested that the majority of patients, regardless of socio-economic background, might require some sort of education on data collection and interpretation and which data would be termed “meaningful”:

It could be with, you know, with some training, maybe somebody on the care team sort of goes through the form back and forth with them a time or two, and make sure they understand the questions and the rating scale, just so that they have a proper level with it. And there may be some where they just not interested or the challenge of trying to complete that form is just beyond their capabilities. (1-06 Sponsor/funder)

Different patients are more or less ready for co-production. So we have to sort of meet patients where they are, so it has to sort of cross the spectrum of patients’ readiness for being involved in their care and…what their values are and what their views are in terms of co-producing as opposed to, you know, being more explicitly guided. (2-04 Site lead)

While it was identified that some clinicians might not see the value in using patient-generated data and might be reluctant to try out new ways of partnering with patients, a more practical barrier was the pressurized context of clinic visits and the risk of data overload.

I think the medical system right now is very rushed, and while this may add efficiencies to those conversations, I’m hoping that it doesn’t to a point where time isn’t allocated, enough time isn’t allocated to have the right conversations at the right time. (1-07 Designer)

Understanding how the program affected clinicians’ workflow was particularly important for its success. Clinicians often had busy schedules and very limited time to see patients. If the program and its associated activities occupied much of their time without demonstrating substantial benefits to both the patient and the clinician, then participants predicted that it was destined to sub-optimal use.

So, there are two aspects to this and I’m going to call them humanware and software. Humanware is just how we organize ourselves and our clinic. Do we create this space to capture the patient’s goals, to address the patient’s goals? Do we have time to discuss them and actually follow through on them? So, that’s one aspect, right? And that doesn’t need technology. It needs, just, you know, workflow reorganization. (2-03 Advisor)

Participants proposed that both patients and clinicians would have to be open to changing their expectations and ways of thinking (and working) about how healthcare is delivered in a clinical environment informed by the values of co-production. Patients would have to learn how to use the application, how to reflect on their experiences, and how to set up and articulate their goals for their care and treatment clearly. From the other side, the clinicians would have to learn how to allow patients to lead the clinical consultation, how to actively help them to formulate the clinical encounter around their goals, and how to best manage any negative emotions that patients might experience, for example, by realising through their data that their health is deteriorating.

So patients need to understand how they can contribute to this co-production of service. And professionals need to understand how they can contribute to this co-production of services, and the relationship of patient and professional itself has to develop a capability for this co-productive work, this interdependent work if in fact we will be successful going forward. (2-02 Advisor)

The assumption was that specifically asking patients about their key concerns and goals would lead to better identification of patients’ needs, which in turn would support a clinical interaction explicitly centered around what the patient sees as important for their care and health.

So I think that the most important feature is actually explicitly asking patients what their questions...you know, their main concern, their main question of priority for the visit, actually being... You know, being explicit in asking the key question. So, you know, what is your main concern and what do you want to get out of this? Because I think that I assumed I knew that, but I never asked it explicitly. (2-11 Pilot Site Leader)

### Design stakeholders’ views of tensions and challenges in implementing the learning system

Participants discussed the possibility of various tensions in implementing the learning system that might arise between the multiple stakeholders, largely seeing them as inevitable but manageable.

I think we all come in with a pretty common purpose of, you know, wanting to improve the quality of life and length of life for people with CF, I think we’re all committed to that. So yeah, I think there are struggles with moments of tension, but ultimately we push through them, because I think we all come in with the same objective. (1-07 Designer)

During the follow-up interviews, the commitment to common purpose and shared vision did not waver, but what was required to deliver it was the focus of some disagreement between stakeholders. Some, for instance, felt that the scope of the project had been too ambitious at the start and accordingly was vulnerable at both a technical and social level.

I think what’s the minimum...what’s the stimulus to really get the two parties to engage in a meaningful interaction and what do the healthcare professionals need, what the patients and families need to feel like they’ve got a stake in the outcome of that interaction? And that could be just simply that they’re able to right at the beginning state what happened...what their agenda items are, clarify the agenda at the beginning of the encounter. I think this first iteration, this first dashboard could probably...something less ambitious could have helped the two parties set the right agenda for the meeting and result in a more productive interaction. (2-01 Program sponsor )

One view was that the realization of the dashboard concept in practice had deviated from the initial design requirements, particularly around usability and relevance for both patients and clinicians. They suggested a form of “scope creep” had shifted the focus of the dashboard.

I think the scope and the aim of the project has significantly broadened to the point where, initially I think we were focused on creating like one page or one screen or one just all-encompassing dashboard of an electronic platform to generate this discussion, and instead it kind of turned into a: how do we get every patient, every parent access to all of the data ever collected on their child or themselves that exists within the national registry. (2-08 Pilot Site Leader)

The breadth of information meant that some stakeholders felt that the dashboard risked obstructing rather than supporting the clinical consultation. Though some information might be important to patients’ day-to-day monitoring, much information not immediately relevant to that day’s appointment might mean that for clinicians at least, the system could lose its appeal and deter them from engaging with the dashboard.

I think that it became too much, so trying to sort of graph every patient symptom, every patient-reported detail, it was too much and it became... It was so big and so much that it became unusable. The platform we were using wasn’t real time, so you didn’t have today’s data there and you only had up till the previous visit’s data, and any discussion really centers around how are things going at today’s appointment, and where is your lung function today? So you absolutely have to have real-time data and that’s crucial. (2-11 Pilot Site Leader)

Lack of inter-operability between the dashboard and patients’ electronic medical records remained another important limitation. In the follow-up interviews, some reported that it took valuable time navigating between registries and databases where patient-reported data were held, posing a practical frustration to the realization of the vision of co-production.

So, it seemed like we went from a very contained and we just create a way to generate discussion and problem-solving as a team in clinic to now how do we give families access to all of this data, and started to generate a website or an application that was incredibly big and cumbersome and very difficult to unify as a clinical tool within the outpatient clinic study." (2-01 Site lead )

It was apparent for many that the wider technological infrastructure that could support the delivery of such a program to its full potential was still missing. Other concerns related to the potential for the system to operate forms of “surveillance” of both clinician and patient “performance.” Participants were concerned, for example, about the extent to which candid conversations could take place if patients were afraid of being judged based on self-care data. Some suggested that patients and clinicians might be tempted to “game” data, to avoid judgement from paternalistic clinicians or managerial systems.

And, when of course when you know people are collecting data for what they perceive as judgement there are all kinds of things that will happen, you know selective reporting, you know people start to do things that, to change the numbers. (1-10 Advisor)

Participants hoped that encouraging openness, refraining from judgment, and using co-production to establish what treatment regimen would be feasible for a particular patient might reduce discomfort with data reporting and diminish incentives to enter inaccurate data. Such issues did not weigh as heavily in the second set of interviews, suggesting perhaps that in practice, the potential benefits of the system outweighed any lingering concerns about surveillance.

## Discussion

Much has been written about the potential of the learning health system concept [29]. Yet, few real-life systems have been described in detail or evaluated. Without evaluative insights into early iterations of such systems, their rich potential might be squandered [11]. This study presents the views of design stakeholders of one promising learning health system and identifies from early experiences where the challenges might lie in moving from design principles to intended outcomes.

The vision and values informing the RCLS-CF—a registry-based learning system for cystic fibrosis—enjoyed a high degree of consensus among the health professional design stakeholders we interviewed, and remained stable between the two phases of interviews (reflected, for example, in an unchanged high-level model—Fig. 1). The founding principle was that, by realizing the principles of co-production [28], a learning system could support patients and clinicians to become confident, competent, and equal partners who can share decisions [30], thus countering the traditional tendency for medical consultations to neglect patients’ views in favor of biomedical understandings and prioritize clinical metrics over patients’ lived experiences [31]. Participants proposed that this kind of learning system would not only generate benefits for patients, but also for research and service improvement.

In practice, the challenges to realizing this vision did not arise from any fundamental disagreement about the underlying objectives or values. Instead, one set of obstacles was, in many ways, quite mundane in character [32]: It involved the technical problems of trying to align systems and data in ways that would be consistent with the strategic vision. Participants pointed, for example, to the need for a parsimonious dataset, not least to avoid drowning in the the ocean of potential insights offered by “big data.” A second set of challenges arose because of difficulties in seeking to refigure traditional forms of clinician-patient relationship. Some of these were seen to be social in character, for example, linked to the discomforts of relinquishing long-established and traditional patient-professional relationships. However, many other problems were seen to arise in seeking to implement changes when time and resource constraints remain real deterrents to shared decision-making [33]. These findings add to the growing body of evidence that highlights highly mundane, practical challenges as a source of trouble in implementing grand visions [32].

A strength of this study was its longitudinal component, gaining insights from stakeholders both before and after implementation of the system. However, a notable weakness was that it did not include patients or patient representatives. Patients’ perspectives on the dashboard might have differed quite substantially from those of clinicians, sponsors, advisors, and designers, and we note in particular that they may have had very different views about the appropriateness of the volume of data included in the dashboard, given the potential usefulness of these data in managing their condition on a day-to-day basis. A further limitation of our study was some attrition between phase 1 and phase 2 interviews. It will be important to determine which of the principles are specific to the CF context and to the US healthcare system and which are generalizable.

## Conclusions

What is important for patients, who must live with and adjust to a chronic condition every day, and what is important for discussion between patients and clinicians in much more sporadic consultations may differ [34]. Developing a learning system that can accommodate both of these needs, as well as serving the purposes of research and service improvement, is not straightforward. Our study has shown that even when the values and vision underlying a learning system are clearly and consistently articulated by design stakeholders, implementation may be challenged by problems that are both social and technical and that may have a strongly mundane character.

## Data Availability

No datasets are available from this study owing to the consents given by participants, which limits data to the research team only.

## Abbreviations

RCLS-CF: Registry-Supported Care and Learning System for people with cystic fibrosis
TiDIER: Template for Intervention Description and Replication
